# Supplementary material for: Pathophysiological Significance of Neutrophilic Transfer RNA-Derived Small RNAs in Asymptomatic Moyamoya Disease
Source: Cells. 2021 May 1;10(5):1086. doi: 10.3390/cells10051086 (PMC8147334; doi:10.3390/cells10051086)

2D Structure	Local AU	Position

```
2578                      8mer-1a     2605
 5'-ctctGTTTTAA-GAAAACAATATGTGGGa-3' utr
        :|::|||    |  | :||||||
 3'-uccuUAGGAUUGGCGAUCUGGUACACCCu-5' tRF
                16151413  765432
              3' Pairing    Seed
```

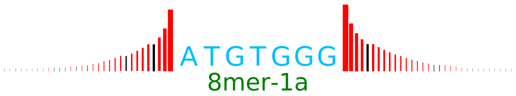

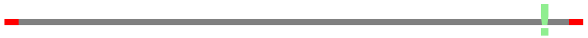

2D Structure	Local AU	Position

```
668                       8mer-1a     696
 5'-gtagtagatacgcaacAGATAATGTGGGa-3' utr
                    |||: ||||||||
 3'-uccuuaggauuggcgaUCUGGUACACCCu-5' tRF
                16151413  765432
              3' Pairing    Seed
```

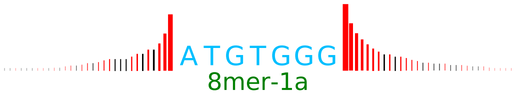

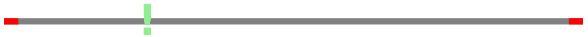

Supplement: Supplementary file 1 [file cells-10-01086-s001.zip › cells-1149161 3rd supplementary materials/Data Supplements/Data Supplement V.zip!/Data supplement 6-mRNAs targets of tsRNAs/tsRNA-Glu-TTC-010/Diagrams/tRF-Glu-TTC-010_vs_ENST00000265138(ARRDC3).pdf]
